# Oculofacial plastic surgery fellowship director perspectives on accreditation council for graduate medical education (ACGME) accreditation

**DOI:** 10.3389/fopht.2026.1867005

**Published:** 2026-07-08

**Authors:** Ayaka Fujihashi, Katerina Tori, Michael M. Han, John B. Holds, Alon Kahana, Steven M. Couch, Hui Bae H. Lee

**Affiliations:** 1 Department of Ophthalmology, Indiana University, School of Medicine, Indianapolis, IN, United States; 2 Oculofacial Plastic and Orbital Surgery, Carmel, IN, United States; 3 Departments of Ophthalmology and Otolaryngology/Head and Neck Surgery, Saint Louis University School of Medicine, St. Louis, MO, United States; 4 Ophthalmic Plastic and Cosmetic Surgery, Inc., St. Louis, MO, United States; 5 Department of Ophthalmology, Oakland University William Beaumont School of Medicine, Royal Oak, MI, United States; 6 Kahana Oculoplastic & Orbital Surgery, Livonia, MI, United States; 7 Barnes-Jewish Hospital, Washington University, St. Louis, MO, United States

**Keywords:** ACGME accreditation, fellowship training, graduate medical education, oculofacial plastic surgery, surgical education

## Abstract

**Introduction:**

Currently, the American Society of Ophthalmic Plastic and Reconstructive Surgery is considering a transition to Accreditation Council for Graduate Medical Education accreditation for oculofacial plastic surgery fellowships. We evaluated fellowship directors’ perspectives and identified perceived benefits and barriers to this change.

**Methods:**

A cross-sectional, national survey was conducted in October 2025 of all American Society of Ophthalmic Plastic and Reconstructive Surgery fellowship directors. A three-question electronic survey assessed support for Accreditation Council for Graduate Medical Education accreditation (yes/no), with predefined response options for opposition and an open-ended field for support.

**Results:**

Forty-four directors completed the survey (83.0%). Nine (20.5%) supported transitioning to Accreditation Council for Graduate Medical Education accreditation, and 35 (79.5%) opposed. Among those opposed, the most frequently cited concerns were the burden of regulation (n=33, 94.3%), duty-hour restrictions (n=29, 82.9%), decreased training (n=23, 65.7%), cost (n=25, 71.4%), limitations on fellows’ billing ability (n=24, 68.6%), and lack of institutional support (n=21, 60.0%). Supporters of the change cited the credibility of Accreditation Council for Graduate Medical Education oversight as the “gold standard” in graduate medical education and the potential pathway toward American Board of Medical Specialties certification.

**Discussion:**

Most fellowship directors oppose transitioning to Accreditation Council for Graduate Medical Education accreditation due to regulatory and financial concerns, while a minority view it as an avenue for enhanced legitimacy and board certification.

## Introduction

Graduate medical education in the United States has undergone significant transformations over the past several decades, largely shaped by the Accreditation Council for Graduate Medical Education (ACGME). The ACGME establishes and enforces national standards for residency and fellowship programs, emphasizing competency-based education, oversight of the learning environment, and the development of well-prepared and independent physicians. One of the most significant changes made by the ACGME was the implementation of duty-hour restrictions in 2003, designed to optimize trainee well-being, education, and patient safety. These regulations limited resident workweeks to 80 hours, mandated a minimum of 10 hours off between shifts, and required one day off per week (1). In 2011, additional regulations limited PGY-1 work hours to 16 consecutive hours with an 8 hour minimum time off period (2).

Within ophthalmology, the American Society of Ophthalmic Plastic and Reconstructive Surgery (ASOPRS) oversees fellowship training in oculofacial plastic surgery—a subspecialty historically independent from ACGME regulation. Recently, ASOPRS leadership has begun exploring ACGME accreditation as a pathway to formal board certification with the American Board of Ophthalmology (ABO). This exploration is not a mandate; participation in ACGME accreditation would remain optional for individual programs. To inform this discussion, we conducted a national survey of all current ASOPRS fellowship directors to evaluate their perspectives, gauge levels or support or opposition, and identify perceived benefits, barriers, and anticipated effects of ACGME accreditation on fellowship training.

## Materials and methods

A brief, three-question survey was developed to assess oculofacial plastic surgery fellowship preceptors’ perspectives regarding the potential transition to ACGME accreditation for ASOPRS fellowships. The survey asked whether respondents supported moving forward with ACGME accreditation with “Yes” or “No” answer options. Respondents who selected “No” were asked to identify reasons from a list of predefined options, including cost, duty-hour restrictions, lack of institutional support, decreased training, burden of ACGME regulations, and the inability of fellows to be able to bill for professional services. Predefined options for reasons opposing ACGME accreditation were developed collaboratively by the study investigators based on recurring themes from prior discussions within the ASOPRS community. A free-text field was provided for additional comments. Respondents who selected “Yes” were prompted to elaborate on their rationale for support within another free-text field. The full survey can be found in **Table 1**. The survey instrument was developed and refined to ensure clarity and content validity. The study was deemed exempt from institutional review board oversight by the Indiana University Institutional Review Board and was conducted in accordance with the tenets of the Declaration of Helsinki.

**Table 1:** Survey instrument assessing fellowship director perspectives on ACGME accreditation.

Question	Question	Response Options
#1	Do you support the decision to move forward with ACGME for your fellowship?	• Yes • No
#2	If no, why?	• Cost • Duty hour restrictions for fellows • Lack of institutional support • Concern for a decrease in training quality • Burden of ACGME regulatory requirements • Concern that fellows will not be able to bill • Other (free text)
#3	If yes, why?	(Open ended free text box)

Three-item survey including a binary support question, predefined reasons for opposition, and an open-ended response for support.

Email addresses of all current ASOPRS fellowship directors were obtained from the publicly available ASOPRS directory. All 53 directors listed in the directory were invited to participate. The survey was distributed in October 2025 via email and with use of the Qualtrics survey platform. The invitation email described the study purpose, stated that participation was voluntary and anonymous, and included a secure link to the online survey. Completion of the survey implied consent. A single reminder email was sent 3 days after initial distribution to maximize participation. The survey remained open for two weeks. No other methods were used to contact program directors.

Responses were collected and stored securely within the Qualtrics system. Quantitative data were summarized using descriptive statistics. Open-ended responses were qualitatively reviewed by two authors to identify recurring themes and representative statements.

## Results

Fifty-three ASOPRS fellowship directors were invited to participate, of which 44 directors completed the survey (83.0%). Nine respondents (20.5%) supported transitioning to ACGME accreditation, and 35 (79.5%) opposed. Among the 35 opposing respondents, the most frequently cited concerns were the burden of ACGME regulation (n=33, 94.3%), duty-hour restrictions (n=29, 82.9%), decreased training (n=23, 65.7%), cost (n=25, 71.4%), limitations on fellows’ ability to bill for professional services (n=24, 68.6%), and lack of institutional support (n=21, 60.0%). Qualitative comments reflected apprehension regarding loss of independence and increasing administrative complexity, as emphasized by the high responses for the predefined options. An additional recurrent theme among opposing the change to ACGME training was the concern over the lack of discussion and transparency from ASOPRS leadership. Supporters of the change to ACGME accreditation cited ACGME’s national credibility, its role as the “gold standard” in graduate medical education, and the potential for eventual American Board of Medical Specialties (ABMS)-recognized board certification. Representative quotes from opposers and supporters of ACGME certification can be seen in **Table 2**. To preserve respondent anonymity, only quotes reflective of the broader themes are included.

**Table 2:** Representative qualitative responses regarding ACGME accreditation.

Do you support the decision to move forward with ACGME for your fellowship?	Representative quotes
“No”	“There is simply not enough information provided.” “…I do not support transitioning at this time without adequate discussion and due diligence.” “ACGME certification adds administrative burden, limits program independence, and will make training fellows financially infeasible for many programs.” “ACGME limits the ability of fellows to act independently.”
“Yes”	“ACGME accreditation, while perhaps burdensome for the PD, is the gold standard in graduate medical education.” “ACGME is not perfect but it is the only way to achieve BC [board certification] that helps us reach the goals of the society.”

Selected free-text responses stratified by support or opposition, illustrating key themes related to administrative burden, autonomy, training, and standardization.

## Discussion

This national survey represents the first assessment of fellowship preceptors’ perspectives on ACGME accreditation within the field of oculoplastics. The 83.0% response rate exceeds the average participation rates for physician-based surveys in ophthalmic plastic surgery literature, where the mean response rate has been reported as 38.7% (range 17.5–60%) across *Ophthalmic Plastic and Reconstructive Surgery* publications (3). Such high engagement underscores the relevance and urgency of this topic among ASOPRS fellowship directors. The majority of ASOPRS fellowship program directors currently oppose transitioning to ACGME accreditation, primarily due to concerns regarding diminished autonomy, regulatory and administrative burden, and cost.

### Duty hours and training

One of the biggest changes made to medical education in the last few decades is the implementation of duty hour restrictions. Though made with the intention of improving patient safety and resident education, studies across medical and surgical specialties have demonstrated mixed results on resident wellness after these changes. A 2023 systematic review found that shorter resident duty hours were associated with improvements in emotional exhaustion, overall well-being, and sleep duration among residents. However, other studies have found that while ACGME reforms reduced reported duty hours, they did not significantly improve sleep, depressive symptoms, or overall well-being scores (4–6). One author has suggested that duty hour restrictions improve resident well-being only when accompanied by a meaningful reduction in workload; reductions in hours without a corresponding decrease in the workload may instead be counterproductive (7). In addition, surveys of program directors in various specialties have consistently shown that duty hour restrictions failed to improve the perceived quality of patient care or resident education while simultaneously increasing administrative burden (8–11). A systematic review on the impact of duty hours on resident education found that the implementation of duty hours was associated with worse quality and safety of care for surgical patients and a significant reduction in continuity of care and educational continuity for residents in surgical subspecialties (12). While no direct mechanism for these worse outcomes was identified in these studies, it can be postulated that increased frequency of provider handoff and the subsequent programmed relinquishing of responsibility could contribute to these worse outcomes.

There is concern that duty hour restrictions may also diminish autonomy and overall clinical exposure for fellows. The concern is particularly great in the field of oculofacial plastic surgery, where trainees are only given two years to master a significant breadth of surgical procedures. The effects of decreased autonomy can be observed among general surgery residents, where the average number of teaching assistant cases performed by general surgery residents markedly decreased in the early 2000s, in part attributed to the implementation of regulations that required the presence of an attending surgeon (13).

### Administrative and financial burden

While the educational impact of ACGME accreditation on fellows is arguably the central consideration of this proposed transition, the equally critical issue of feasibility—namely the administrative and financial burdens placed on preceptors and programs—cannot be overlooked. As currently mandated by ACGME, duties of the program director include but are not limited to: competence assessment, annual program evaluation, maintenance of program policies, duty hour monitoring, curriculum development, and appropriate documentation of said responsibilities for ACGME reporting (14). Time to complete said tasks must be protected as granted by the sponsoring institution. In the field of nephrology, program directors must spend an average of 20 hours per week on administrative duties related to accreditation (15). Though this time can be negotiated individually between the institution and the program leadership, there is no denying its impact on preceptors, ultimately resulting in less time in clinic or the operating room. The ever-growing burdens of being a program director result in a shift from mentorship to management as increasingly prescriptive accreditation requirements generate extensive work that may not meaningfully improve education, reduce clinical productivity, and may not be fairly compensated. Even if these duties are delegated to administrative staff, these staff must be paid, which increases the total cost of training the fellow. These demands have been cited as a factor in burnout among anesthesiology program directors (16). Implementation of duty hour restrictions has also led to the transfer of patient care responsibilities to faculty. One study found that as a result of resident work hour limitations, faculty spend more time on clinical work, felt more responsible for supervising patient care, and spent less time on research or other academic pursuits including teaching residents (17). In a small, highly specialized field such as oculofacial plastic surgery, in which most programs rely on a few key faculty members, the cumulative effects of increased administrative demands and redistributed clinical responsibilities are magnified, placing disproportionate strain on preceptors and threatening the sustainability of fellowship training itself.

Financially, ACGME accreditation poses further challenges for ASOPRS fellowship programs due to the administrative and institutional infrastructure required for compliance. Large academic centers may already have much of this infrastructure in place, whereas these requirements may create a disproportionate burden for smaller academic or private practice-based fellowships. Indeed, the cost of compliance can be quite staggering. Kempenich et al. estimated that the annual cost of compliance for an average general surgery residency program is $227,043, with ACGME-mandated costs constituting $218,143 of the total. This was largely due to labor costs of the program director, faculty time spent teaching (including participation in lectures, morbidity & mortality conferences, journal clubs, etc.), and the program coordinator’s salary (19). Similarly, in the field of internal medicine, the fixed instructional and administrative costs mandated by ACGME requirements were predicted to exceed $640,000 per year for program administration costs and mandated instructional activities (20).

In contrast, ASOPRS fellowships have historically operated with greater flexibility and without equivalent accreditation-related administrative requirements, allowing training opportunities in a broader range of settings with qualified preceptors. Fellowship compensation under the current ASOPRS models is also variable across programs and may derive from multiple sources, including institutional salary support, faculty practice revenue, fellow billing, Veterans Affairs clinical responsibilities, or on-call coverage (18).

### Potential benefits: ACGME accreditation and board certification

Pursuing ACGME accreditation for oculofacial plastic surgery fellowships can address training standardization, institutional credentialing, trainee protections and professional advocacy. ACGME accreditation establishes a national recognized framework for graduate medical education that ensures programs meet standards in curriculum design, clinical, and surgical volume, and trainee evaluation. Formal ACGME accreditation of fellowship training may become increasingly consequential as hospital systems expect ACGME-accredited training for privileging in hopes to address rising concerns of public accountability for patient safety, quality of care, and efficiency in delivery of care (21).

Several respondents support ACGME accreditation as a means for enhanced legitimacy, institutional recognition, and a pathway to ABMS certification. Interestingly, even among respondents that support this change, many acknowledge the significant burdens that would be associated with it. It is important to contextualize these concerns, however. More than 80 medical specialties and subspecialties—including all major recognized disciplines in medicine—currently operate within the ACGME framework, encompassing nearly 14,000 accredited programs and over 162,000 trainees as of 2024-2025 (22). These programs span a wide range of sizes, settings, and resource levels, and have successfully navigated many of the same regulatory, administrative, and financial challenges cited by respondents in our survey. While the unique characteristics of oculofacial plastic surgery training—including the small program size, reliance on individual preceptors, and two-year training duration—may amplify certain burdens, the concerns identified here are not unique to this subspecialty and have been addressed by the broader graduate medical education community through institutional support structures, administrative delegation, and iterative adaptation to ACGME requirements.

This tension mirrors the experience of other surgical subspecialties. In pediatric otolaryngology, 64% of fellowship graduates agreed that standardizing fellowship training through ACGME accreditation was a worthwhile goal, and ACGME-accredited program graduates reported tangible benefits including greater protected research time, more adequate vacation and academic time, and lower call burden (23). These potential benefits are particularly relevant given the significant heterogeneity across ASOPRS fellowship programs in surgical volume, case diversity, and program characteristics. ACGME accreditation could provide standardized trainees protections, duty hour regulations, and formal evaluation mechanisms that could address this variability.

The decision by ASOPRS leadership to explore board certification is distinct from questions surrounding ACGME accreditation of individual programs. Seeking board certification does not necessarily imply that ACGME accreditation will be required for all fellowship programs. While ACGME-accredited training is typically a prerequisite for ABMS certification and eligibility, the specific pathway and requirements for any future board certification in ophthalmic plastic surgery would be determined through negotiations between ASOPRS, the relevant ABMS member board, The American Board of Ophthalmology, and the ACGME. Notably, board certification has practical implications on obtaining surgical privileges and malpractice coverage. This study was designed to assess fellowship directors’ perspectives on ACGME accreditation specifically; it does not attempt to evaluate attitudes toward or the merits of ABMS board certification, which warrants its own dedicated investigation.

ASOPRS fellowship directors demonstrate a strong interest in the future of their training structure, as evidenced by the exceptionally high participation rate in our survey. Our findings emphasize the necessity for further discussion, engagement, and communication from ASOPRS leadership with fellowship preceptors before moving forward with a decision that will have broad and significant implications for the structure and quality of fellowship training.

This study has several limitations. First, because participation was voluntary, the findings may be influenced by response bias, as fellowship directors with stronger opinions about a potential transition to ACGME accreditation—particularly those with concerns or opposition—may have been more likely to respond. The survey’s brevity, while intended to maximize response rates, limited the depth of analysis and may not have captured the full range of perspectives or nuances of respondents’ opinions. The survey questions, which utilized predetermined options for those opposing the change and free-text options for those supporting the change, may have introduced asymmetry in how perspectives were expressed, potentially biasing responses toward certain themes. However, because respondents were free to select multiple options or provide additional comments, the structured format likely facilitated clarity and comparability without meaningfully constraining expression of viewpoints. Additionally, the respondents’ view of this issue is evolving as they learn more about ACGME program requirements as well as how their existing program might adapt to accommodate those requirements. We believe that the discussion related to ACGME accreditation of ASOPRS fellowships has broader relevance to medical training, particularly since the costs and administrative burdens of healthcare and medical training continue to increase, while the relative funds and resources supporting these activities continue to decrease. Finally, we note that several authors of this study- Dr. Holds, Dr. Kahana, and Dr. Lee- are current ASOPRS fellowship directors who would be directly affected by changes to accreditation requirements. While fellowship directors possess firsthand knowledge of the practical realities of training and are therefore well-positioned to identify operational barriers, their perspectives should be interpreted alongside those of other stakeholders whose views were not captured in this survey. We present these data not as a definitive position on accreditation but as one important contribution to an ongoing, multistakeholder dialogue.
